# Gabapentin in Acute Postoperative Pain Management

**DOI:** 10.1155/2014/631756

**Published:** 2014-04-14

**Authors:** Connie Y. Chang, Chaitanya K. Challa, Janki Shah, Jean Daniel Eloy

**Affiliations:** Department of Anesthesia, Rutgers New Jersey Medical School, 185 South Orange Avenue, MSB E-538, Newark, NJ 07101-1709, USA

## Abstract

Gabapentin (1-aminomethyl-cyclohexaneacetic acid) is an amino acid that has the structure of the neurotransmitter **γ**-aminobutyric acid (GABA). It is a novel drug used for the treatment of postoperative pain with antihyperalgesic properties and a unique mechanism of action. Gabapentin and the related, more potent compound pregabalin have been shown to be beneficial in the treatment of neuropathic pain as well as postoperative pain following spinal surgery and hysterectomy. This study reviews five aspects of gabapentin: (1) chemical and structural characteristics; (2) pharmacokinetics and pharmacodynamics; (3) application in acute pain management; (4) adverse effects; and (5) drug safety. Overall, gabapentin has been reported to be a safe and efficacious drug for the treatment of postoperative pain.

## 1. Introduction

Primarily, three different classes of drugs are utilized for the treatment of postoperative pain (anti-inflammatories, local anesthetics, and opioids). Unfortunately long-term clinical use of these agents is limited by their side effects. Gabapentin is a novel drug used for the treatment of postoperative pain with antihyperalgesic properties and a unique mechanism of action, which differentiates it from other commonly used drugs. Various studies have shown that perioperative use of gabapentin reduces postoperative pain.

Gabapentin works by reducing lesion-induced hyperexcitability of posterior horn neurons, which is responsible for central sensitization [1]. The mechanism of the antihyperalgesic action may be a result of the postsynaptic binding of gabapentin to the alpha_2_-delta subunit of the dorsal horn neurons' voltage-dependent calcium channels, causing decreased calcium entry into nerve endings and thus decreased release of neurotransmitters. Other possible cellular mechanisms include the effects of gabapentin on NMDA receptors, sodium channels, monoaminergic pathways, and the opioid system [2–5].

Gabapentin was initially introduced in 1994 as an antiepileptic drug (AED), primarily for partial seizures. It is an anticonvulsant whose side effects are well tolerated and well absorbed after oral administration with the maximal plasma concentration seen after two to three hours [2, 6]. Some of the most commonly reported side effects of gabapentin include dizziness, somnolence, fatigue, ataxia, and peripheral edema [4, 7].

Gabapentin has also been found to be beneficial in treating neuropathic pain related to postherpetic neuralgia (PHN) [8, 9], postpoliomyelitis neuropathy [10], and reflex sympathetic dystrophy [11]. Additionally, gabapentin has been shown to play a role in treating pain related to diabetic neuropathy [12] in placebo-controlled clinical trials.

This study reviews five aspects of gabapentin: (1) chemical and structural characteristics; (2) pharmacokinetics and pharmacodynamics; (3) application in acute pain management; (4) adverse effects; and (5) drug safety.

## 2. Chemical and Structural Characteristics

Gabapentin [1-(aminomethyl)cyclohexaneacetic acid] is a structural analog of the inhibitory neurotransmitter gamma-aminobutyric acid (GABA) [13]. It is a white to off-white crystalline solid substance with the molecular formula, C_9_H_17_NO_2_, and a molecular weight of 171.237 g/mol. Freely soluble in water and in both basic and acidic aqueous solutions, gabapentin is highly charged at physiological pH and exists as a zwitterion with a p*K*_a1_ of 3.68 and p*K*_a2_ of 10.70 [14]. Gabapentin is assayed in plasma and urine using gas chromatography [15], high performance liquid chromatography [16], and high performance liquid chromatography with UV-vis detection [17].

## 3. Pharmacodynamics

Though it is structurally similar to GABA, gabapentin does not bind to GABA_*A*_ or GABA_*B*_ receptors, does not block GABA uptake or metabolism, and has no direct GABAergic action [18]. The mechanism of action of gabapentin is different from that of several drugs that interact with GABA synapses such as valproate, barbiturates, and benzodiazepines; it is thought to exert its action by interaction with a receptor associated with the L-system amino acid carrier protein. In vitro studies with radiolabelled gabapentin have shown that gabapentin binds preferentially to neurons in the outer layer of the rat cortex and the hippocampus at sites distinct from other anticonvulsants [19]. Because the maximal anticonvulsant effect is seen 2 hours after an intravenous injection in rats, it is probable that gabapentin exerts its action at an intracellular site [20].

Gabapentin inhibits the tonic phase of nociception stimulated by formalin and carrageenan and in neuropathic pain models prevents mechanical and thermal allodynia and mechanical hyperalgesia [18]. Though the mechanism of action of gabapentin in the treatment of neuropathic pain is not clear, it does not influence the same pathways as opioids or tricyclic depressants. Current evidence indicates that gabapentin affects voltage-gated calcium channels in the CNS.

Gabapentin binds to the *α*_2_ *δ* subunit of the voltage-dependent calcium channel, regulating the action of the calcium channels and neurotransmitter release [15, 21, 22]. It is suggested that the antihyperalgesic action of gabapentin is due to its binding to this site on the voltage-gated calcium channel. Fink et al. demonstrated that gabapentin blocks neuronal calcium influx in a concentration-dependent manner by inhibiting P/Q-type calcium channels in the rat neocortex [23]. Decreased AMPA receptor activation and norepinephrine release in the brain are observed due to the reduced calcium influx, which decreases excitatory amino acid (glutamate) release [24]. These results support the hypothesis that the analgesic effects of gabapentin in neuropathic pain are mediated by the inhibition of voltage-gated calcium channels.

Other effects of gabapentin that do not have major pharmacodynamic significance include slight reductions in the release of monoamine neurotransmitters (dopamine, norepinephrine, and serotonin) from mammalian brain tissue [25, 26] and a decrease in sodium-dependent action potentials, indicating sodium channel blockade, after prolonged exposure [27].

## 4. Pharmacokinetics

Administered orally, gabapentin is absorbed in part by diffusion and in part by the carrier-mediated, L-amino acid transport system [28]. As a result of the saturable transport mechanism, the bioavailability of gabapentin is inversely dependent on the dose [29]. It ranges from approximately 60% for a 300 mg dose [30] to 40% for a 600 mg dose [31] and 35% at steady state with doses of 1600 mg three times daily [32]. The bioavailability of gabapentin is not affected by the presence of food and remains unchanged following multiple dose administration. Mean maximum plasma gabapentin concentrations (*C*_max⁡_) of 2.7 ± 2.99 mg per liter are reached in healthy volunteers approximately 3 hours after a single oral 300 mg dose [30, 33]. When the dose is tripled from 300 mg to 900 mg, *C*_max⁡_ increases less than threefold due to the dose dependent saturable absorption of gabapentin [32].

Gabapentin has a high volume of distribution of 0.6–0.8 L/kg or 50–60 L in healthy volunteers [30, 32, 33] and does not bind to human plasma proteins [34]. Unlike GABA, gabapentin readily penetrates the blood-brain barrier, yielding CSF concentrations equal to 20% of plasma concentrations and estimated between 0.09 and 0.14 *μ*g per mL [33, 35]. Brain tissue concentrations are 80% of corresponding plasma levels [36]. In rats, gabapentin concentrates in the pancreas and kidneys. Pancreatic and renal tissue concentrations are eight and four times higher than serum concentrations, respectively [37]. The drug does not accumulate in the pancreas in humans since it exists in a highly ionized state at physiological pH and concentrations in adipose tissue are low [37].

In contrast to many antiepileptic drugs, which are metabolized, gabapentin is not metabolized in humans and is eliminated solely by renal clearance. The drug is excreted unchanged in urine and undergoes first order kinetic elimination [29, 33]. Plasma clearance of gabapentin is directly proportional to creatinine clearance; consequently, renal impairment reduces gabapentin excretion and increases plasma gabapentin concentrations in a linear fashion [33, 34, 37, 38]. Patients with renal failure should receive their maintenance dose of gabapentin after each treatment as gabapentin is removed during hemodialysis [39]. Although a dose response pattern is apparent for plasma gabapentin concentrations and for clinical effect for doses ranging from 600 to 1800 mg per day, it is not necessary to monitor plasma gabapentin concentrations and gabapentin dose should be modified based on clinical response [33]. The elimination half-life of gabapentin is between 5 and 9 hours, and as a result, three divided doses are usually required per day, but steady state is rapidly achieved [29].

Gabapentin is unique among anticonvulsant drugs as it lacks hepatic metabolism, exhibits low protein binding, and does not induce or inhibit hepatic microsomal enzymes or inhibit the metabolism of other antiepileptic drugs [29, 34]. No significant pharmacokinetic interactions have been reported between gabapentin and conventional antiepileptic drugs (valproic acid, phenobarbital, carbamazepine, or phenytoin) or oral contraceptives [29, 34]. However, cimetidine, which decreases glomerular filtration rate, reduces the clearance of gabapentin by 12% [32]. In addition, the bioavailability of gabapentin is decreased by 20% by antacids when taken simultaneously or up to 2 hours after gabapentin administration [40].

## 5. Application in Acute Pain Management

### 5.1. Coronary Artery Bypass Graft

Ucak et al. evaluated the analgesic effects of perioperative gabapentin after coronary artery bypass graft (CABG) surgery with median sternotomy as well as internal mammary artery harvesting [41]. They used a gabapentin dose of 1.2 g per day treatment 1 hour before surgery and for 2 days after surgery and investigated its effect on postoperative acute pain. In this study, postoperative pain scores at 1, 2, and 3 days as well as the consumption of tramadol which was given as rescue analgesic were significantly lower in the gabapentin group when compared to the placebo group [41].

### 5.2. Thoracic Surgery

Zakkar et al. performed a literature search and identified five papers with the best evidence regarding the use of gabapentin to reduce the incidence of pain experienced by patients after thoracic surgery [42–46]. They concluded that there is no evidence to support the role of a single preoperative oral dose of gabapentin in reducing pain scores or opioid consumption after thoracic surgery. Furthermore, more robust randomized control studies are needed to validate the efficacy of multiple dosing regimens but studies currently show that it may be beneficial in reducing acute pain [42].

### 5.3. Thyroid Surgery

Lee et al. explored the efficacy of using gabapentin (600 mg) 1 hour before the administration of anesthesia for thyroid surgery [47]. He determined that the gabapentin group had a lower incidence of postoperative sore throat (POST) and a significantly lower visual analogue scale (VAS) score at 6 and 24 hours at rest after the completion of the surgery compared to the placebo group. However, there was no intergroup difference between the gabapentin group and the placebo group in terms of the incidence of POST or VAS score during the swallowing movement. From this study they were able to conclude that the gabapentin administered 1 hour prior to anesthesia decreased the intensity and incidence of POST at rest without a significant adverse event within the first 24 hours after thyroid surgery but not during the swallowing movement [47].

### 5.4. Neurological Surgery

Misra et al. performed a study to investigate patients undergoing craniotomies and the efficacy of gabapentin plus dexamethasone on postoperative nausea and vomiting (PONV) and pain after craniotomy [48]. Patients undergoing craniotomy received gabapentin (600 mg) premedication orally 2 hours prior to induction of anesthesia as well as 4 mg of intravenous dexamethasone on the morning of surgery and continued receiving it after every 8 hours. The 24-hour incidence of nausea, emesis, or PONV and postoperative pain scores were evaluated. This study observed a significant difference between the group that received gabapentin and dexamethasone and the placebo group in the incidence of nausea and the requirements for antiemetics. However, there was no significant difference in either the postoperative pain scores or the opioid consumption between the gabapentin with dexamethasone cohort and the placebo cohort. Therefore, although there was no reduction in either the postoperative pain scores or opioid consumption, gabapentin plus dexamethasone significantly reduced the 24-hour incidence of nausea and PONV [48].

### 5.5. Lumbar Spinal Surgery

Yu et al. performed a systematic review and meta-analysis to determine the efficacy of gabapentin in the management of postoperative pain after lumbar spinal surgery. They showed that oral gabapentin was efficacious in the management of postoperative pain at every time point during the first day after surgery and therefore is efficacious in reducing postoperative pain and narcotic requirements after lumbar spinal surgery [49].

### 5.6. Hysterectomy

Ajori et al. performed a study investigating the preemptive use of gabapentin (600 mg) prior to abdominal hysterectomy and its influence on nausea and vomiting, and meperidine consumption [50]. Pain was assessed on a visual analogue scale (VAS) at 1, 4, 6, 12, and 24 hours postoperatively. This study showed that gabapentin group had significantly lower VAS scores at every time interval compared to the placebo group and the total meperidine consumed in the gabapentin group was significantly less than in the placebo group. PONV and the consumption of antiemetic drugs were also significantly reduced in the gabapentin group. Therefore, preemptive use of 600 mg gabapentin orally in patients undergoing abdominal hysterectomies significantly decreases postoperative pain and PONV and also reduces analgesic and antiemetic drug requirements [50].

### 5.7. Major Bowel Surgery

Siddiqui et al. performed a study investigating the effects of gabapentin (600 mg) orally 1 hour prior to surgery in patients with inflammatory bowel disease (IBD) undergoing major bowel surgery. This group found that a single preoperative administration of 600 mg gabapentin in patients undergoing major bowel surgery does not reduce postoperative pain scores, opioid consumption, or opioid-related side effects [51].

### 5.8. Orthopedic Surgery

Panah Khahi et al. explored the efficacy of preemptive use of gabapentin on reduction of postoperative pain in the first 24 hours after internal fixation of the tibia under spinal anesthesia [52]. Patients were administered 300 mg of gabapentin two hours before surgery and postoperative pain was evaluated using VAS two, 12 and 24 hours after surgery. The time from the completion of the surgery until the first bolus dose of morphine on demand and the total morphine required were also evaluated. This study showed that the pain score was significantly lower in the gabapentin group compared to the placebo group two hours after the completion of the surgery; however, the scores 12 and 24 hours after surgery were not significantly different between the two groups. Therefore, preemptive use of gabapentin 300 mg orally significantly alleviated postoperative pain two hours after internal fixation of the tibia [52].

## 6. Adverse Effects

Gabapentin may have dose-limiting side effects, which could prevent some patients from achieving therapeutic plasma levels. Studies have reported no significant difference in the frequency of adverse events in patients receiving a higher dose as compared to those receiving a lower dose of gabapentin [53]. The most consistently cited adverse effects of gabapentin are somnolence and dizziness. While these side effects are considered to be minor, they may be worrisome in the elderly population, which is prone to injuries from falls and gait instability. Given this issue, clinicians may opt to discontinue gabapentin in clinical practice in this age group [54]. One possible explanation for these side effects is the multiple peaks and troughs in plasma concentration levels observed with treatment regimens consisting of multiple doses per day [54]. Since many clinical trials have shown that gabapentin demonstrates improvement in sleep, there may be an advantage for a formulation of gabapentin that peaks during the night, with lower levels during the day.

Clivatti et al. performed a study looking at twenty-six randomized, placebo-controlled, clinical studies between 2005 and 2007 and evaluated the effects of gabapentin preoperatively (PRE Group) and pre- and postoperatively (PRE-POST Group) [55]. The study reported adverse effects which included postoperative nausea and vomiting, sedation, and dizziness. In the PRE Group, which consisted of seventeen studies, a reduction in the incidence of nausea and vomiting in patients treated with gabapentin was observed in two studies while an increase in nausea and vomiting was reported in one study. Additionally, a higher incidence of sedation was reported in one study while a higher incidence of dizziness was seen in another study. In the PRE-POST Group, which consisted of nine studies, one study reported an increase in the incidence of postoperative nausea and vomiting, another study reported an increase in sedation, and two studies reported an increase in the incidence of dizziness [55]. Likewise, a study by Fassoulaki et al. did not observe intolerable side effects with the use of gabapentin for the daily dose of 1600 mg/d. Some sedation was exhibited by their patients, especially in the beginning of treatment, but that is desirable before and immediately after surgery.

Turan et al. investigated the effects of gabapentin on acute postoperative pain and on morphine consumption in patients undergoing spinal surgery where 1,200 mg gabapentin was given 1 hour before surgery. The most common adverse effects of gabapentin observed during the study were dizziness and nausea but the number of incidences did not significantly differ from the placebo group and therefore no significant adverse effects were associated with a single oral dose of gabapentin [56]. However, in that same study, there was a significant decrease in vomiting and urinary retention in the gabapentin group versus the placebo group; therefore gabapentin decreased the side effects associated with morphine in patients undergoing spinal surgery [56].

Sen et al. showed that although the most common side effects after an elective hysterectomy were nausea and vomiting, there were no differences in the incidence of these side effects between the perioperative administration of a placebo, ketamine, and gabapentin [57]. Similarly, Gilron et al. assessed the efficacy and tolerability of gabapentin, nortriptyline, or a combination of both which showed that, both during dose titration and at the maximum tolerated dose, the most common adverse event was moderate or severe dry mouth, which occurred significantly less frequently in patients on gabapentin than on nortriptyline or combination treatment [58]. There were no other significant differences in adverse events [58].

Furthermore, a study by Compton et al. evaluated the efficacy of gabapentin, a key agent for neuropathic pain, to reverse opioid-induced hyperalgia in methadone maintenance for the treatment of addiction patients [59]. The two most commonly reported adverse events in this study were nausea and dizziness/lightheadedness which were both reported to be greater in the gabapentin group than in the placebo group [59].

A major complication that develops after the abrupt discontinuation of high-dose gabapentin used for the prevention of chronic postsurgical pain is withdrawal symptoms that clinically resemble alcohol or benzodiazepine withdrawal [60]. Signs and symptoms include irritability, agitation, anxiety, palpitation, and diaphoresis within 1-2 days. Secondary to gabapentin withdrawal, a patient with chronic back pain also developed generalized seizures and status epilepticus [61]. Tapering should always be performed especially in patients taking higher doses; however, withdrawal syndrome can still rarely be observed despite dose tapering [62].

## 7. Pharmacovigilance

Gabapentin has been reported to be a well-tolerated and safe drug [12, 33, 63]. Studies on safety issues have reported adverse effects including dizziness, somnolence, confusion, headache, nausea, ataxia, and weight gain [53, 64]. However, these side effects were usually reported after long-term gabapentin use and usually diminish with time but may be bothersome in an acute setting to a postoperative patient. Studies which reported these side effects used the drug for 8 weeks to doses titrated up to 3600 mg per day, unless severe adverse effects were developed.

Rowbotham et al. conducted a randomized controlled trial to determine the safety of gabapentin in reducing postherpetic neuralgia [63]. In this study, they used 229 subjects and performed a 4-week titration period to maximum dose of 2600 mg per day of gabapentin or matching placebo. The treatment was maintained for another 4 weeks at the maximum tolerated dose. They found that minor adverse events that were determined to be associated with the study medication were reported in 62 subjects receiving gabapentin and 32 subjects receiving placebo. The investigator reported no serious adverse events that were related to gabapentin. In this study, the most frequently reported adverse effects within the gabapentin group which were greater than the placebo group were somnolence (27.4% versus 5.2%), dizziness (23.9% versus 5.2%), ataxia (7.1% versus o.o%), peripheral edema (9.7% versus 3.4%), and infection (8.0% versus 2.6%) [63].

McLean et al. performed a study which evaluated and demonstrated the tolerability and safety of gabapentin when used as an adjunctive therapy in doses required to achieve the most effective seizure control in patients with epilepsy [53]. This study compared tolerability of gabapentin dosages less than or equal to 1,800 mg versus those greater than 1,800 mg per day. The safety analysis required patients to be followed up after receiving at least one dose of gabapentin and included 2,216 patients [53]. The safety of gabapentin therapy was evaluated by the following parameters: (1) adverse event reports, (2) physicians' assessment of safety and tolerability at study completion or termination, (3) neurologic examinations at baseline and study completion or termination, and (4) vital signs (sitting systolic and diastolic blood pressure, sitting heart rate) which were taken at baseline and study completion or termination. An adverse event was defined as a noxious or unintended event observed in or reported by a patient who was participating in the study and had received study medication and these adverse events were further classified relative to gabapentin therapy as definitely, probably, possibly, unlikely/remotely, or definitely not related. An associated adverse event was defined as an event that was considered by the investigator to be definitely, probably, or possibly related to treatment with gabapentin.

In this study, McLean et al. showed that 48.3% of patients reported the occurrence of at least one adverse event and 36.4% of patients reported at least one associated adverse event [53]. The most common adverse events for patients receiving less than or equal to 1,800 mg/day were asthenia, headache, dizziness, and somnolence while the most common adverse events for patients receiving greater than 1,800 mg/day were somnolence, dizziness, and weight gain. No adverse events were reported to occur at a significantly higher percentage of patients while receiving the higher dose compared to the lower dose of gabapentin. The rate of withdrawal from the study did not increase at higher doses suggesting that the incidence of adverse events does not increase at higher doses or longer use of gabapentin [53].

A serious adverse event was defined as any event that was fatal, life-threatening, and permanently disabling, required or prolonged inpatient hospitalization, or was a congenital anomaly, cancer, or overdose and was reported in only 3.3% of patients [53]. The most common serious adverse event was convulsion, reported by 0.9% of patients [53]. Eleven (0.4%) patients died during the study although no deaths were considered to be associated with gabapentin treatment [53]. The safety and tolerability of gabapentin as determined by the physicians at the completion of the study or dropout were judged to be good or excellent for 78.5% of patients. Additionally, the treatment of gabapentin did not adversely affect vital signs in this study [53]. Overall, gabapentin was determined to be safe and doses greater than 1,800 mg/day were well tolerated and not associated with more adverse events.

Backonja et al. investigated the effect of gabapentin monotherapy on pain associated with diabetic peripheral neuropathy by titrating from 900 to 3600 mg per day or maximum tolerated dosage or placebo [12]. The safety of gabapentin was determined by using adverse event data (occurrence, intensity, and relationship to gabapentin) and the results of physical and neurological examination, including peripheral sensory examinations. During this study a total of seven gabapentin-treated patients (8%) withdrew from the study due to adverse events which included dizziness and somnolence, abdominal pain, asthenia, body odor, headache, diarrhea, abnormal thinking, nausea, confusion, and hypesthesia. Out of the placebo group, only 5 patients (6%) withdrew due to dyspepsia, constipation, flatulence infection, and somnolence. Most adverse events in patients treated with gabapentin were described as mild or moderate intensity. There were no significant changes in hemoglobin A_1c_ levels from baseline to the termination of treatment in either group. As such, glycemic control was maintained during the study. Neurological examination data revealed no significant differences in the rate of disease progression and the proportion of patients with a change from normal or decreased at baseline to absent at study termination was similar between groups (less than 14% in each case) for the 3 sensory modalities tested (temperature, light touch, and pin prick). Dizziness and somnolence were the two adverse events reported to occur more frequently in the gabapentin group. Overall, this study showed that gabapentin monotherapy produced rapid onset of clinical meaningful pain relief with relatively minor and potentially avoidable adverse effects; in turn, this further supports the safety of gabapentin as described in the prior studies [12].

## 8. Conclusion

Clinical studies have shown that gabapentin can reduce acute postoperative pain, decreasing the need for opioids. For example, a gabapentin dose of 1.2 grams per day 1 hour before surgery and for 2 days after CABG surgery showed that postoperative pain scores at 1, 2, and 3 days as well as the consumption of tramadol given as a rescue analgesic were significantly lower in the gabapentin group when compared to the placebo group [41]. Additionally, preemptive use of gabapentin 300 mg orally significantly alleviated postoperative pain two hours after internal fixation of the tibia. Similarly, preemptive use of 600 mg gabapentin orally in patients undergoing abdominal hysterectomies significantly decreases postoperative pain and POVN and also reduces analgesic and antiemetic drug requirements [50]. While some studies suggest that gabapentin does not significantly reduce postoperative pain scores or opioid consumption, it has been shown to significantly reduce the 24-hour incidence of nausea and PONV. Overall, gabapentin has been reported to be a well-tolerated, safe, and efficacious drug.
